# Supplementary material for: Leveraging SO(3)-steerable convolutions for pose-robust semantic segmentation in 3D medical data
Source: J Mach Learn Biomed Imaging. Author manuscript; Available in PMC 2024 Dec 9. (PMC7617181; doi:10.59275/j.melba.2024-7189)
Supplement: Supplementary Material [file EMS201157-supplement-Supplementary_Materials.pdf]

Philip Müller, Vladimir Golkov, Valentina Tomassini, and Daniel Cremers. Rotation-equivariant deep learning for diffusion mri. *arXiv preprint arXiv:2102.06942*, 2021.

Shuchao Pang, Anan Du, Mehmet A Orgun, Yan Wang, Quanzheng Sheng, Shoujin Wang, Xiaoshui Huang, and Zhemei Yu. Beyond cnns: Exploiting further inherent symmetries in medical images for segmentation. *arXiv preprint arXiv:2005.03924*, 2020.

Olaf Ronneberger, Philipp Fischer, and Thomas Brox. U-net: Convolutional networks for biomedical image segmentation. In *International Conference on Medical image computing and computer-assisted intervention*, pages 234–241. Springer, 2015.

Nathaniel Thomas, Tess Smidt, Steven Kearnes, Lusann Yang, Li Li, Kai Kohlhoff, and Patrick Riley. Tensor field networks: Rotation-and translation-equivariant neural networks for 3d point clouds. *arXiv preprint arXiv:1802.08219*, 2018.

Dmitry Ulyanov, Andrea Vedaldi, and Victor Lempitsky. Instance normalization: The missing ingredient for fast stylization. *arXiv preprint arXiv:1607.08022*, 2016.

Maurice Weiler, Mario Geiger, Max Welling, Wouter Boomsma, and Taco S Cohen. 3d steerable cnns: Learning rotationally equivariant features in volumetric data. *Advances in Neural Information Processing Systems*, 31, 2018.

Jim Winkens, Jasper Linmans, Bastiaan S Veeling, Taco S Cohen, and Max Welling. Improved semantic segmentation for histopathology using rotation equivariant convolutional networks. In *International Conference on Medical Imaging with Deep Learning*, 2018.

Jin Xu, Hyunjik Kim, Thomas Rainforth, and Yee Teh. Group equivariant subsampling. *Advances in Neural Information Processing Systems*, 34:5934–5946, 2021.

7. Appendix

Radial Basis Functions

Since equivariance to rotation implies factorization of the kernel into a radial and angular component, the radial component has to be parameterized. These functions are chosen to be smooth and go to zero at the cutoff radius. To enable learning of parameters, we characterise the radial function as a sum of smooth basis elements. The equation is given by:

$$8.433573\ sus(x+1)sus(1-x) \tag{3}$$

with sus (soft unit step) defined as follows:

$$sus(x) = \begin{cases} e^{-1/x} & x > 0 \\ 0 & x \leq 0 \end{cases}$$

Equation 3 is a C^{∞} function and is strictly zero for x outside the interval $[-1, 1]$. The prefactor 8.433573 ensures proper normalization of the neural network and was obtained empirically.

Rotation results up to 20° around the three different planes

In the following figures, we show the dice performance of the rotation-equivariant model G-CNN models and reference network with no data-augmentation, with data augmentation up to 20° and full data augmentation on the test set rotated through angles from 0° to 20°.

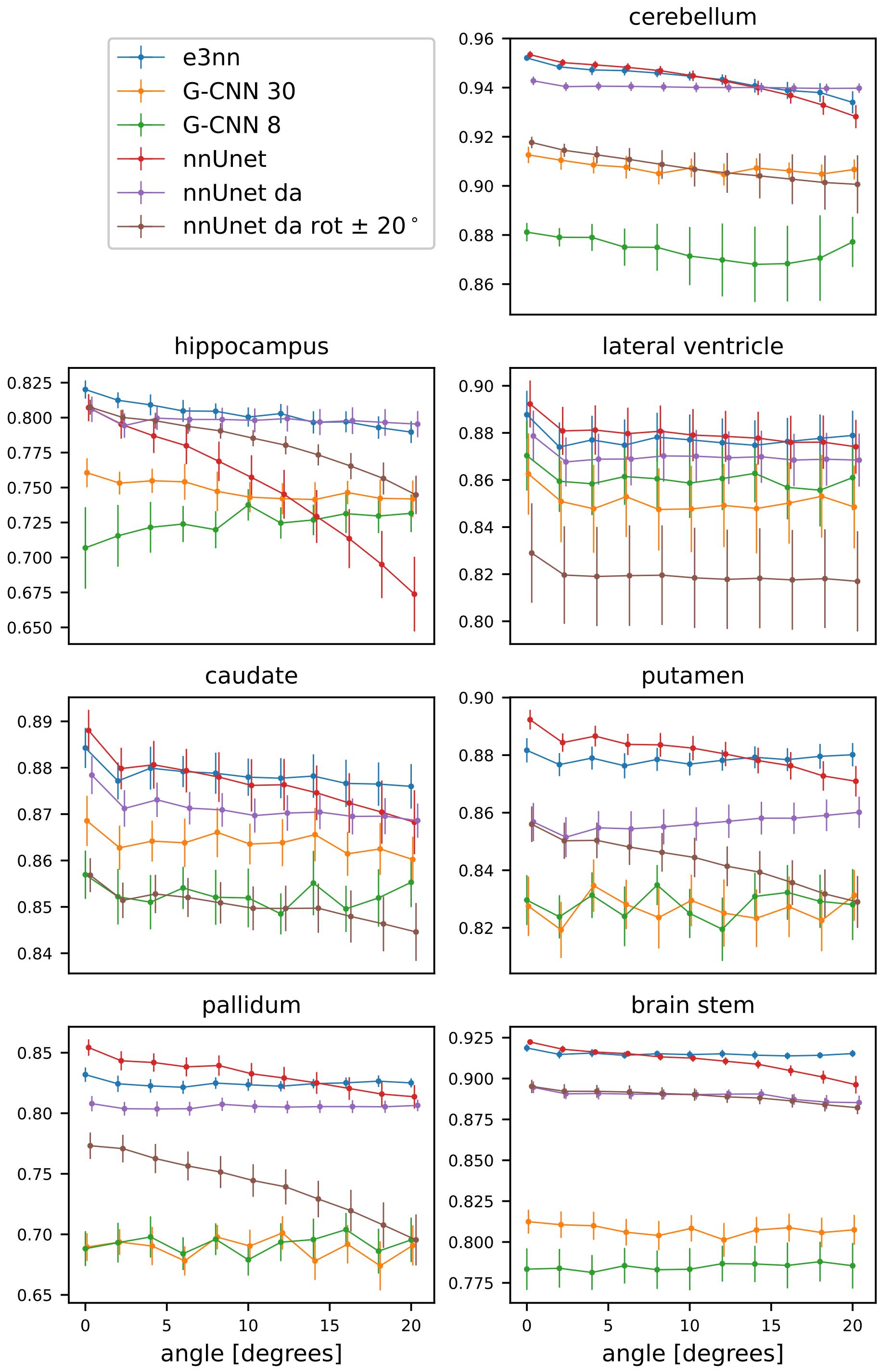

Figure 7: Dice score on the test set vs rotation angle in the saggital plane for seven brain structures. The error bars display the uncertainty on the mean estimator of the average dice score across the 10 samples in the test set.

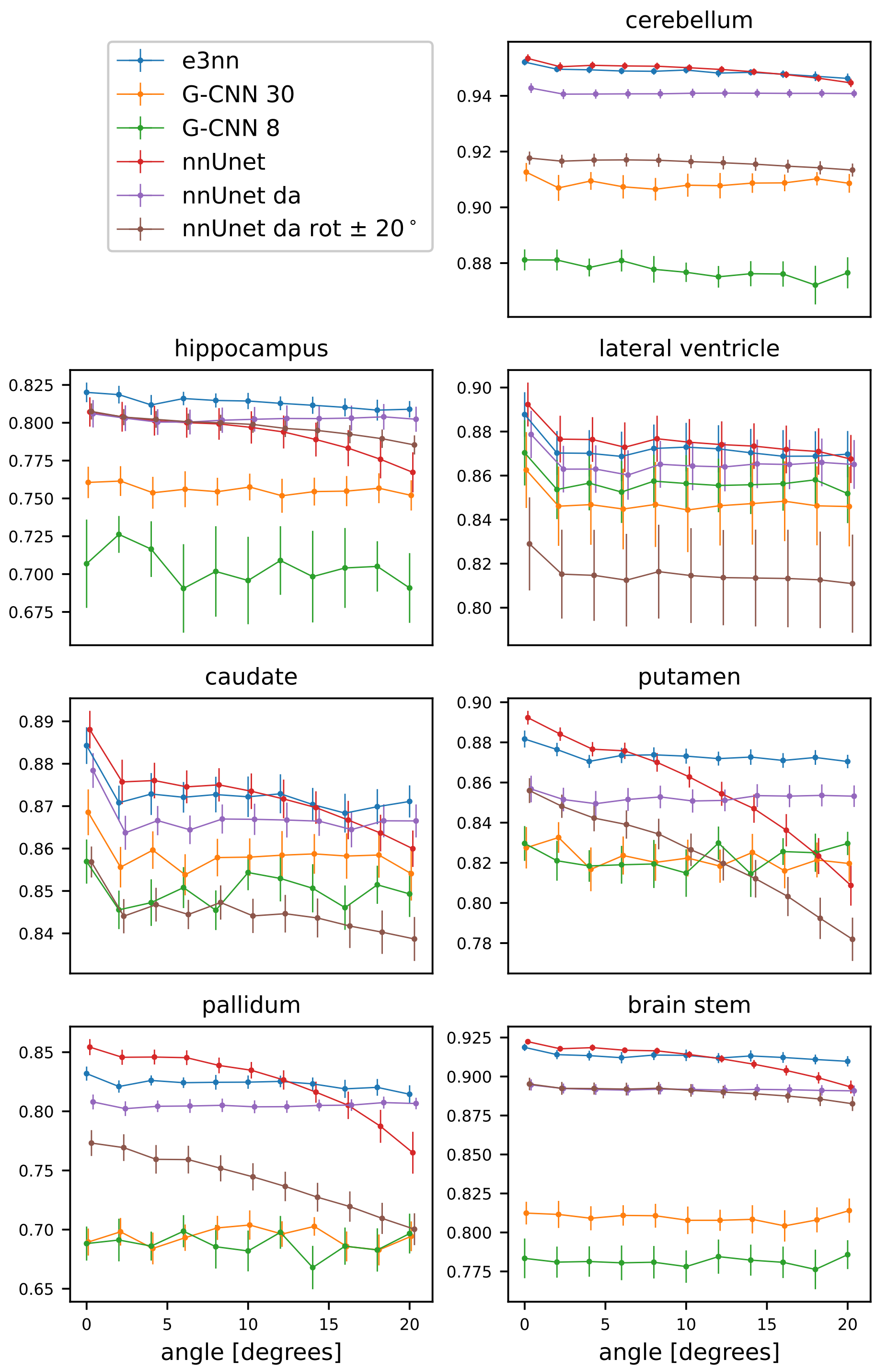

Figure 8: Dice score on the test set vs rotation angle in the coronal plane for seven brain structures.

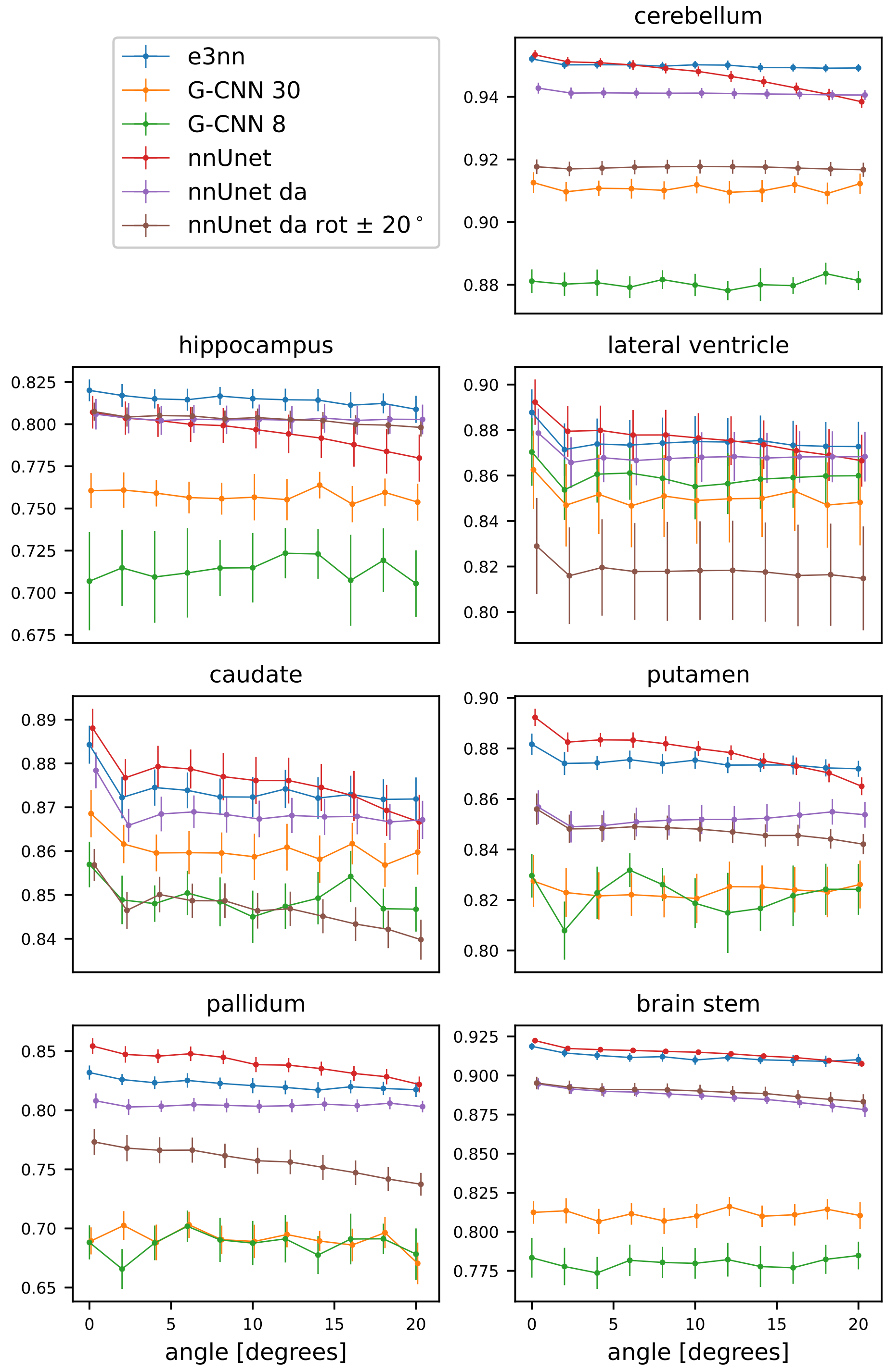

Figure 9: Dice score on the test set vs rotation angle in the axial plane for seven brain structures.
